# Neoadjuvant Treatment is a Risk Factor for Clinically Relevant Chyle Leak (ISGPS Grade B/C) After Pancreatic Cancer Resection: A Retrospective Cohort Study

**DOI:** 10.1245/s10434-025-18698-4

**Published:** 2025-11-21

**Authors:** Carl-Stephan Leonhardt, Sakher Shraim, Karim Abdelazim, Ulf Hinz, Georgios Polychronidis, Thomas Hank, Martin Schneider, Thilo Hackert, Oliver Strobel, Martin Loos, Markus W. Buechler, Mohammed Al-Saeedi

**Affiliations:** 1 Department of General, Visceral, and Transplantation Surgery, University of Heidelberg, Heidelberg, Germany; 2 Department of General Surgery, Division of Visceral Surgery, Medical University of Vienna, Vienna, Austria; 3 Department of General, Visceral, Thoracic and Transplantation Surgery, University Hospital Giessen, Giessen, Germany; 4 Botton-Champalimaud Pancreatic Cancer Center, Champalimaud Foundation, Lisbon, Portugal; 5 General, Visceral and Thoracic Surgery Department and Clinic, University Medical Center Hamburg-Eppendorf, Hamburg, Germany

**Keywords:** Pancreatic ductal adenocarcinoma, Neoadjuvant treatment, Pancreatic surgery, Chyle leak, ISGPS classification

## Abstract

**Background:**

Chyle leak is a relevant complication after surgery for pancreatic ductal adenocarcinoma (PDAC). Increasing use of multimodal neoadjuvant therapy (NAT) and extended resections may contribute to a higher incidence. This study aimed to describe the incidence of clinically relevant chyle leak and identify its risk factors in patients with PDAC treated with NAT and surgery versus upfront surgery.

**Patients and Methods:**

Patients undergoing PDAC resection between 2014 and 2019 were identified from a prospective institutional database. Clinicopathologic and demographic data were extracted. The primary outcome was clinically relevant chyle leak according to the International Study Group of Pancreatic Surgery (ISGPS). Univariable and multivariable logistic regression were performed to identify associated factors.

**Results:**

A total of 1402 patients were included: 1090 (77.7%) underwent upfront surgery, and 312 (22.3%) received NAT. Pancreatic head resections were performed in 718 (51.2%), distal pancreatectomies in 317 (22.6%), and total pancreatectomies in 367 (26.2%) individuals. Overall, 108 (7.7%) developed clinically relevant chyle leak: 88 (6.3%) grade B and 20 (1.4%) grade C. NAT (odds ratio [OR] 1.45, 95% confidence interval [CI] 1.10–1.91), T stage (pT2/3/4 versus pT0/1, OR 1.53, 95% CI 1.03–2.34), and total pancreatectomy (OR 2.29, 95% CI 1.78–2.96) were associated with any chyle leak. For grade B/C, only NAT was an independent risk factor (OR 2.63, 95% CI 1.74–3.94). Age, resection type, and nodal status were not independent risk factors.

**Conclusions:**

NAT is an independent risk factor for clinically relevant chyle leak following PDAC resection. These findings highlight the need for risk-adapted postoperative management and warrant prospective validation.

**Supplementary Information:**

The online version contains supplementary material available at 10.1245/s10434-025-18698-4.

Chyle leak is a common postoperative complication after pancreatic surgery, due to damage to lymphatic vessels during resection and lymphadenectomy.^1^ Reported incidence rates in literature vary widely, ranging from 1% to 22%, depending on the definition used, the extent of surgery, and institutional practices.^2^ A previous study from our center reported an incidence of 10.4% after upfront resection.^3^ Importantly, chyle leak leads to clinically relevant consequences, including delayed recovery, prolonged hospitalization, nutritional deficiency, and interruption of oncologic therapy.^4,5^ Notably, while most chyle leaks resolve with conservative management such as dietary modification (medium-chain triglyceride diet), total parenteral nutrition (TPN), and maintenance of drains, some necessitate additional interventions, including therapeutic lymphography.^1,5–7^

Several risk factors for chyle leak have been identified. First, the extent of surgery has been associated with a higher risk of chyle leak, with more radical procedures such as extended lymphadenectomy, para-aortic lymphadenectomy, total pancreatectomy, and vascular resection posing greater risk.^2,6,8,9^ In addition, open surgery, pre-existing diabetes, distal pancreatectomy, and resection for malignancy were associated with a higher risk of chyle leak in retrospective series.^3,10^ To facilitate comparability and further research, the International Study Group of Pancreatic Surgery (ISGPS) proposed a standardized definition and classification system, dividing chyle leaks into grade A (asymptomatic or self-limiting), grade B (requiring interventions such as dietary modification or drainage), and grade C (associated with major morbidity).^11^

Recently, the management of pancreatic ductal adenocarcinoma (PDAC) has shifted toward a multimodal approach. In particular, for patients with borderline resectable (BR) or locally advanced tumors (LAPC), neoadjuvant treatment (NAT) is now widely used to improve resectability, reduce micrometastatic disease, and potentially improve long-term outcomes.^12–16^ However, owing to fibrosis and scarring of tissue, NAT may obscure anatomical planes and increase the risk of lymphatic injury during surgery, potentially contributing to a higher incidence of chyle leak.^17,18^

On the basis of these considerations, we hypothesized that NAT is associated with an increased risk of clinically relevant chyle leak (ISGPS grade B/C) after resection for PDAC. Thus, the aim of this study was to describe the incidence of clinically relevant chyle leak in patients undergoing resection with and without neoadjuvant treatment for PDAC and identify potential risk factors.

## Patients and Methods

### Study Design and Population

This retrospective, single-center cohort study was conducted at the Department of General, Visceral, and Transplantation Surgery at the University Hospital Heidelberg and followed the Strengthening the Reporting of Observational Studies in Epidemiology (STROBE) guidelines.^19^ The study was approved by the institutional ethics committee (approval no. S-619/2024), and patient consent was waived owing to the retrospective nature of the analysis.

All consecutive patients with histologically confirmed PDAC who underwent pancreatic resection between January 2014 and December 2019 were eligible for inclusion. Consistent with the study hypothesis, patients were divided on the basis of preoperative treatment strategy into patients who received NAT prior to surgery and patients who underwent upfront surgery.

### Data Collection and Variables

Data were extracted from a prospectively maintained institutional database. Additional information was extracted from the electronic health record. Demographic, clinicopathologic, and outcome variables were collected. Chyle leak was graded according to the ISGPS definition.

On the basis of domain knowledge, the following variables besides NAT were considered as potential risk factors of chyle leak: type of surgery performed (pancreatoduodenectomy, distal pancreatectomy, or total pancreatectomy), the extent of resection (type 1–4),^20^ tumor stage, lymph node status, duration of surgery, and amount of blood loss during the operation. No data on race/ethnicity were available as these are not collected routinely within the German healthcare system.

### Outcome Measures

The primary outcome was the occurrence of clinically relevant chyle leak (ISGPS B/C). Secondary outcomes included perioperative morbidity and overall survival.

### Statistical Analysis

Continuous variables are presented as medians or means, with interquartile ranges (IQR) and standard deviations, as appropriate, and were compared using the Mann–Whitney *U* test. Categorical variables are reported as counts and percentages and compared using the chi-squared test or Fisher’s exact test, as appropriate. Univariable logistic regression analyses were conducted to identify factors associated with clinically relevant chyle leak (grade B/C). Variables with a *p*-value < 0.1 in the univariable analysis were included in the multivariable logistic regression model. Adjusted odds ratios (aORs) with 95% confidence intervals (CIs) were reported. Missing data were assessed for each variable; if applicable, variables with > 5% missing data were excluded from multivariable analysis. No information on neoadjuvant treatment regimens was available for two patients, which were subsequently excluded from the analysis. Survival was analyzed using the Kaplan–Meier method from time of surgery, and differences were assessed using the log-rank test. All statistical analyses were performed using SAS software (Release 9.4, SAS institute, Inc., Cary, NC, USA), and a two-sided *p*-value < 0.05 was considered significant.

## Results

### Patient Characteristics

**Fig. 1:**
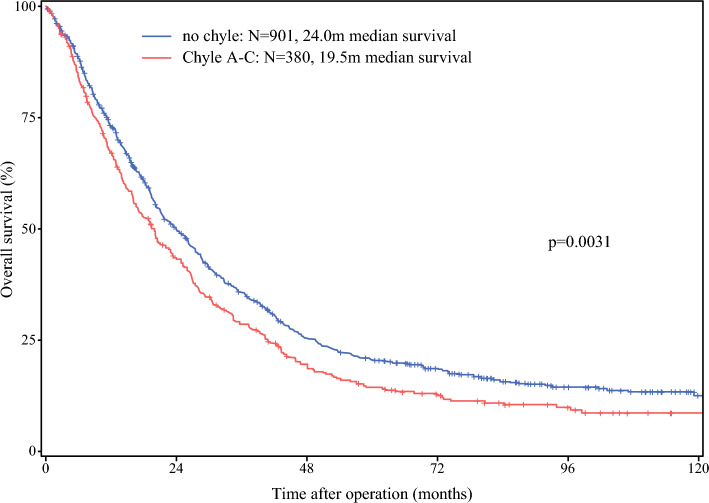
Overall survival for the combined cohort stratified by any type of postoperative chyle leak versus no chyle leak. Log-rank test was used for comparison

A total of 1402 patients who underwent pancreatic resection for PDAC between 2014 and 2019 were included (Table 1). Of these, 312 patients (22.2%) received NAT, while 1090 (77.8%) underwent upfront surgery. Within the NAT group, 247 of 310 patients (79.7%) received FOLFIRINOX, and 33 patients (10.6%) received gemcitabine/nab-paclitaxel. Patients in the NAT group were younger, with a median age of 61.9 years (IQR 53.8–67.8), compared with 68.3 years (IQR 60.5–75.3) in the upfront surgery group (*p* < 0.0001). More extensive surgical procedures were performed in the NAT group, including total pancreatectomy in 111 of 312 patients (35.6%) versus 256 of 1090 (23.5%), and type 4 vascular resections in 67 of 312 (21.5%) versus 42 of 1090 (3.8%) in the upfront group (*p* < 0.0001 for both). Median intraoperative blood loss was 1300 mL in the NAT group and 800 mL in the upfront group (*p* < 0.0001), and the duration of surgery was 360 versus 312 min, respectively (*p* < 0.0001). In total, 121 patients (8.6%) were lost to follow-up with a median follow-up time of 20.1 months (IQR 9.3–41.9). In the combined cohort, patients who developed any kind of chyle leak had a shorter median overall survival compared with those without chyle leak (19.5 versus 24.0 months, *p* **=** **0.003**) (Fig. 1). Consistently, median overall survival was shorter in upfront resected patients who developed any kind of chyle leak compared with no chyle leak (20.1 versus 26.2 months, Supplementary Fig. 1). In contrast, among patients who received NAT followed by resection, median survival did not significantly differ between those with and without a chyle leak (17.1 versus 19.5 months, *p* = 0.89) (Supplementary Fig. 2).

**Table 1:** Patient characteristics

Median (IQR), *n* (%)	Total	PDAC with neoadjuvant CTx	PDAC without neoadjuvant CTx	*P*-value
	(*n* = 1402)	(*n* = 312)	(*n* = 1090)	
Demographic data				
Age (years)	66.3 (58.8–74.1)	61.9 (53.8–67.8)	68.3 (60.5–75.3)	**< 0.0001**
BMI, kg/m^2^	24.7 (22.2–27.2)	23.9 (21.7–26.3)	24.9 (22.5–27.5)	**< 0.0001**
Sex				
Female	672 (47.9)	151 (48.4)	521 (47.8)	0.85
Male	730 (52.1)	161 (51.6)	569 (52.2)	
Preoperative data				
Diabetes				
Yes	339 (25.0)	74 (26.0)	265 (25.0)	0.75
No	1005 (75.0)	211 (74.0)	794 (75.0)	
Missing	58	27	31	
Jaundice				
Yes	539 (46.5)	76 (34.2)	463 (49.5)	**< 0.0001**
No	619 (53.5)	146 (65.8)	473 (50.5)	
Missing	244	90	154	
ASA classification				
I	65 (4.9)	10 (3.3)	55 (5.4)	0.48
II	761 (57.4)	170 (56.9)	591 (57.6)	
III	493 (37.2)	118 (39.5)	375 (36.5)	
IV	6 (0.5)	1 (0.3)	5 (0.5)	
Missing	77	13	64	
Intraoperative data				
Type of surgery				
PD	718 (51.2)	113 (36.2)	605 (55.5)	**< 0.0001**
TP	367 (26.2)	111 (35.6)	256 (23.5)	
DP	317 (22.6)	88 (28.2)	229 (21.0)	
Type of resection				
1	654 (46.6)	75 (24.0)	579 (53.1)	**< 0.0001**
2	359 (25.6)	75 (24.0)	284 (26.1)	
3	280 (20.0)	95 (30.5)	185 (17.0)	
4	109 (7.8)	67 (21.5)	42 (3.8)	
Operation time (min)	320 (248–402)	360 (280–450)	312 (243–390)	**< 0.0001**
Blood loss (mL)	900 (500–1500)	1300 (700–2000)	800 (500–1300)	**< 0.0001**
Histopathological data				
Tumor size status (8th edition)				
pT0	12 (0.9)	12 (3.9)	0 (0.0)	**< 0.0001**
pT1	138 (9.8)	43 (13.8)	95 (8.7)	
pT2	756 (53.9)	156 (50.0)	600 (55.1)	
pT3	466 (33.2)	84 (26.9)	382 (35.0)	
pT4	30 (2.1)	17 (5.4)	13 (1.2)	
Tumor size (cm)	3.5 (2.5–4.4)	3.4 (2.3–4.2)	3.5 (2.5–4.5)	**0.007**
Lymph node status				
pN0	328 (23.4)	123 (39.4)	205 (18.8)	**< 0.0001**
pN1	487 (34.7)	101 (32.4)	386 (35.4)	
pN2	587 (41.9)	88 (28.2)	499 (45.8)	
Number of examined lymph nodes	30 (23–38)	29 (21–38)	30 (23–39)	**0.13**
Number of positive lymph nodes	3 (1–6)	1 (0–4)	3 (1–7)	**< 0.0001**
Lymph node ratio	0.09 (0.02–0.20)	0.04 (0.0–0.12)	0.11 (0.03–0.22)	**< 0.0001**
LNR category				
0	328 (23.4)	123 (39.4)	205 (18.8)	**< 0.0001**
> 0 to < 0.1	413 (29.5)	93 (29.8)	320 (29.4)	
0.1 to < 0.2	307 (21.9)	52 (16.7)	255 (23.4)	
≥ 0.2	354 (25.3)	44 (14.1)	310 (28.4)	
Distant metastasis				
cM0	1278 (90.9)	251 (80.4)	1022 (93.8)	**< 0.0001**
pM1	129 (9.1)	61 (19.6)	68 (6.2)	
Postoperative complications				
Pancreatic fistula				
No/BL	1254 (89.4)	281 (90.1)	973 (89.3)	0.45
B	108 (7.7)	20 (6.4)	88 (8.1)	
C	40 (2.9)	11 (3.5)	29 (2.7)	
Reoperation				
No	1210 (86.3)	253 (81.1)	957 (87.8)	**0.002**
Yes	192 (13.7)	59 (18.9)	133 (12.2)	
PPH				
No	1300 (92.7)	283 (90.7)	1017 (93.3)	0.12
Yes	102 (7.3)	29 (9.3)	73 (6.7)	
Hospital mortality				
No	1358 (96.9)	302 (96.8)	1056 (96.9)	0.94
Yes	44 (3.1)	10 (3.2)	34 (3.1)	
Hospital stay (days)	13 (10–20)	14 (10–22)	13 (9–20)	**0.031**
Chyle leak				
Grade 0	995 (71.0)	199 (63.8)	796 (73.0)	**< 0.0001**
Grade A	299 (21.3)	69 (22.1)	230 (21.1)	
Grade B	88 (6.3)	33 (10.6)	55 (5.1)	
Grade C	20 (1.4)	11 (3.5)	9 (0.8)	
Grade B/C	108 (7.7)	44 (14.1)	64 (5.9)	**< 0.0001**

*PDAC* pancreatic ductal adenocarcinoma, *CTx* chemotherapy, *IQR* interquartile range, *BMI* body mass index, *ASA* American Society of Anesthesiologists, *PD* pancreatoduodenectomy, *TP* total pancreatectomy, *DP* distal pancreatectomy, *pT0–pT4* pathological tumor stage (AJCC 8th edition), *pN0–pN2* pathological nodal status, *cM0* no clinical distant metastasis, *pM1* pathological distant metastasis, *LNR* lymph node ratio, *BL* biochemical leak, *PPH* post-pancreatectomy hemorrhage

### Incidence and Severity of Chyle Leak

**Fig. 2:**
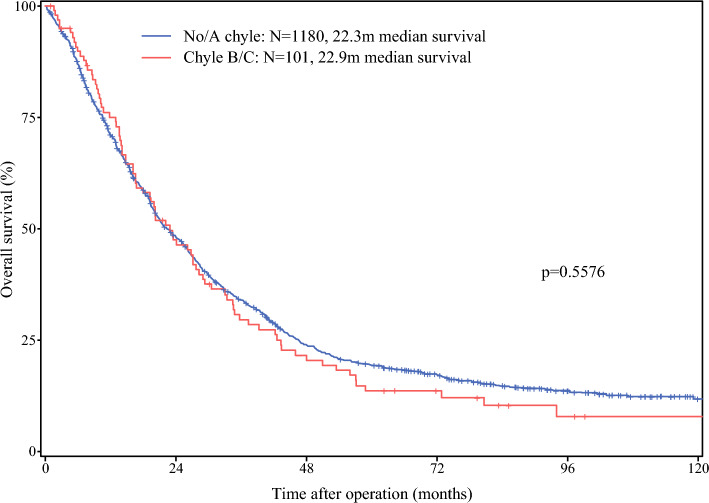
Overall survival for the combined cohort stratified into no/type A chyle leak versus clinically relevant chyle leak (ISGPS grade B/C). Log-rank test was used for comparison

The overall incidence of clinically relevant chyle leak was 7.7% (*n* = 108), with 6.3% (*n* = 88) of patients experiencing grade B, and 1.4% (*n* = 20) grade C. Patients treated with NAT had higher rates of grade B/C chyle leak compared with those undergoing upfront surgery (44 of 312 [14.1%] versus 64 of 1090 [5.9%], *p* < 0.0001). No significant associations were observed between the type of neoadjuvant chemotherapy regimen (FOLFIRINOX versus gemcitabine-based) and chyle leak (*p* = 0.95) (Supplementary Table 1). Patients with grade B/C chyle leak had a median overall survival of 22.9 months, which was comparable to the 22.3 months in those without or with only grade A leak (*p* = 0.56) (Fig. 2).

### Risk Factors of Any Kind of Chyle Leak

On univariable logistic regression analysis, several variables were associated with an increased risk of developing chyle leak. Specifically, NAT was associated with an increased risk of chyle leak (OR 1.54; 95% CI 1.18–2.01). Among surgical procedures, total pancreatectomy versus distal pancreatectomy (OR 2.21; 95% CI 1.60–3.07) and type 4 versus type 1 resections were associated with an increased risk of chyle leak (OR 2.18; 95% CI 1.43–3.31). In contrast, age above 70 years was associated with a lower risk compared with younger patients (OR 0.77; 95% CI 0.60–0.97) (Supplementary Table 2).

### Risk Factors of Clinically Relevant Chyle Leak (Grade B/C)

Next, we specifically examined potential risk factors associated with ISGPS grade B/C chyle leak, consistent with our study aim. In the unadjusted analysis, NAT was significantly associated with clinically relevant chyle leak (OR 2.63; 95% CI 1.74–3.94) (Table 2). On multivariable analysis NAT was an independent risk factor of clinically relevant chyle leak (OR 2.63; 95% CI 1.74–3.94) (Table 3). In the adjusted analysis for any kind of chyle leak, NAT remained an independent risk factor of chyle leak, with an odds ratio of 1.45 (95% CI 1.10–1.91) (Table 3). In addition, total versus distal pancreatectomy and pT2-4 versus pT0-1 were independently associated with any kind of chyle leak (OR 2.29; 95% CI 1.78–2.96 and OR 1.53; 95% CI 1.03–2.34, respectively).

**Table 2:** Univariable analysis of factors associated with chyle leak grade B/C

Parameter	*N*	Chyle leak grade B/C (%)	Odds ratio	95% confidence interval	*P*-value
Total	1402	108 (7.7)			
Age (years)					0.34
< 70	861	71 (8.2)	1	–	
≥ 70	541	37 (6.8)	0.82	0.54–1.23	
Sex					0.40
Male	730	52 (7.1)	1	–	
Female	672	56 (8.3)	1.19	0.80–1.76	
ASA					0.50
ASA I/II	826	68 (8.2)	1	–	
ASA III/IV	499	36 (7.2)	0.87	0.56–1.31	
Diabetes					0.91
No	1005	79 (7.9)	1	–	
Yes	339	26 (7.7)	0.97	0.60–1.52	
Jaundice					0.44
No	619	50 (8.1)	1	–	
Yes	539	37 (6.9)	0.84	0.54–1.30	
Neoadjuvant treatment					**< 0.0001**
No	1090	64 (5.9)	1	–	
Yes	312	44 (14.1)	2.63	1.74–3.94	
Type of surgery					0.45
DP	317	27 (8.5)	1	–	
PD	718	49 (6.8)	0.79	0.49–1.30	
TP	367	32 (8.7)	1.03	0.60–1.76	
Type of resection					0.26
1	654	51 (7.8)	1	–	
2	359	20 (5.6)	0.70	0.40–1.17	
3	280	27 (9.6)	1.26	0.76–2.04	
4	109	10 (9.2)	1.19	0.56–2.34	
Tumor size status					0.81
pT0	12	2 (16.7)	1	–	
pT1	138	12 (8.7)	1	–	
pT2	756	55 (7.3)	0.76	0.42–1.46	
pT3	466	36 (7.7)	0.81	0.44–1.60	
pT4	30	3 (10.0)	1.08	0.24–3.59	
Positive node count					0.51
0	328	24 (7.3)	1	–	
1–3	487	43 (8.8)	1.23	0.74–2.09	
≥ 4	587	41 (7.0)	0.95	0.57–1.63	
Lymph node ratio					0.82
0	328	24 (7.3)	1	–	
> 0 to < 0.1	413	34 (8.2)	1.14	0.66–1.98	
0.1 to < 0.2	307	26 (8.5)	1.17	0.66–2.10	
≥ 0.2	354	24 (6.8)	0.92	0.51–1.66	

*ASA* American Society of Anesthesiologists, *DP* distal pancreatectomy, *PD* pancreatoduodenectomy, *TP* total pancreatectomy, *pT0–pT4* pathological tumor stage (AJCC 8th edition), *LNR* lymph node ratio

**Table 3:** Multivariable analysis of factors associated with any kind of chyle leak (grade A–C) and clinically relevant chyle leak (B/C)

Parameter	Category	Odds ratio	95% confidence interval	*P*-value
Grade A–C				
Type of surgery	TP versus PD/DP	2.29	1.78–2.96	**< 0.0001**
Neoadjuvant chemotheray	Yes versus no	1.45	1.10–1.91	**0.008**
Tumor size status (8th edition)	pT2/3/4 versus pT0/1	1.53	1.03–2.34	**0.04**
Grade B/C				
Neoadjuvant treatment	Yes versus no	2.63	1.74–3.94	**< 0.0001**

*TP* total pancreatectomy, *PD* pancreatoduodenectomy, *DP* distal pancreatectomy, *pT0–pT4* pathological tumor stage (AJCC 8th edition)

## Discussion

NAT is being increasingly used in the treatment of PDAC. Here, we demonstrate that NAT is an independent risk factor of clinically relevant chyle leak (ISGPS grade B/C) following pancreatic resection for PDAC. In addition, patients who underwent NAT were more than twice as likely to develop any kind of chyle leak, even after adjusting for potential confounders such as extent of surgery and tumor stage.

While literature on NAT-associated lymphatic complications is limited, our findings highlight the need to consider chyle leak as a relevant side effect of NAT. NAT is associated with distinct molecular remodeling mechanisms of pancreatic cancer cells and the tumor microenvironment.^21^ These may include cytotoxic damage to peritumoral lymphatics, chemotherapy-induced fibrosis, and impaired healing responses in treated tissues. In addition, NAT results in activation of fibroblasts, and extensive deposition of extracellular matrix components, leading to increased stromal fibrosis and desmoplasia.^17,18^ This fibrotic remodeling can alter normal tissue architecture, obscure anatomical planes, and potentially affect the integrity of lymphatic vessels.

Similar findings have been reported in cervical cancer, where NAT was independently associated with increased lymphatic leakage following pelvic lymphadenectomy.^22^ In adult patients with testicular cancer undergoing retroperitoneal lymph node dissection, increased exposure to neoadjuvant chemotherapy and greater intraoperative blood loss have been identified as a risk factor of postoperative chylous ascites.^23^ Finally, NAT has been identified as a risk factor for postoperative chyle leak after esophagectomy in esophageal cancer in some studies, but the evidence remains inconclusive.^24,25^

Notably, patients with chyle leak (A–C) had poorer overall survival compared with those without chyle leak. However, when the analysis was limited to clinically relevant chyle leaks (grade B/C) compared with no chyle leak or chyle leak grade A, no significant difference in survival was observed. Several factors could account for this. First, it may be attributed to the inclusion of a more heterogeneous patient cohort in the overall survival analysis, potentially encompassing a higher proportion of patients with more advanced tumor stages or other unfavorable baseline characteristics. In addition, consistent with the previous literature, the total share of chyle leak grade B/C amounted to 7.7%, most likely resulting in an insufficient sample size to detect any significant differences in unadjusted Kaplan–Meier survival analysis. Potentially, in a larger cohort, relevant differences in overall survival could be observed. Of note, Strobel et al. report a decreased survival in patients who underwent exploration and resolution of chyle leak ≥ 14 days compared with those with resolution < 14 days while no difference was detected for patients who underwent resection.^3^

An important aspect of our analysis is the distinction between any-grade chyle leak and clinically relevant grade B/C leak. While several variables were associated with overall leak rates, including type of surgery and extent of resection, only NAT remained a significant risk factor of grade B/C chyle leaks in the multivariable model. This is important to acknowledge as grade B/C chyle leak might lead to a change in clinical management, including delayed initiation of adjuvant treatment.^3^

However, several limitations must be acknowledged. First, the use of neoadjuvant therapy was primarily administered to patients who were initially staged as borderline resectable or locally advanced disease. Thus, these patients underwent more extensive resections, potentially contributing to a higher risk of chyle leak independent of an effect of NAT itself. While in our model we tried to adjust for pathological T stage, and type of surgery, residual confounding cannot be excluded in retrospective data. Similarly, it should be acknowledged that T stage and NAT are likely collinear, and therefore, resulting odds ratios should be interpreted with caution as they may not reflect unbiased effect estimates. Studies investigating the effect of neoadjuvant treatment in upfront resectable pancreatic cancer, ideally within a randomized controlled trial to minimize residual confounding, are needed to further elucidate the impact of neoadjuvant treatment on clinically relevant chyle leak.

This is a retrospective, single-center study from a tertiary referral center, which limits generalizability. In addition, selection bias cannot be excluded. While we used standardized definitions and adjusted for key confounders, including extent of surgery based on previous reports, unmeasured variables may have influenced the results. In this regard, data on nutritional status, intraoperative lymphatic control techniques, and postoperative management protocols were not available and could impact leak incidence. In addition, clinical management of chyle leak could not be evaluated in our study on the basis of the retrospective design.

## Conclusions

To the best of our knowledge, this is the first study that examines the association of chyle leak with NAT in patients with PDAC. NAT was an independent risk factor of clinically relevant chyle leak (ISGPS grade B/C) following PDAC resection. Given the increasing use of NAT, heightened awareness and tailored surgical approaches are warranted to mitigate this risk. Prospective validation of these findings is required.

## Supplementary Information

Below is the link to the electronic supplementary material.

Supplementary file1 (DOCX 25 KB)

Supplementary file2 (TIFF 1602 KB)

Supplementary file3 (TIFF 1609 KB)
