# Supplementary material for: Neoadjuvant Treatment is a Risk Factor for Clinically Relevant Chyle Leak (ISGPS Grade B/C) After Pancreatic Cancer Resection: A Retrospective Cohort Study
Source: Ann Surg Oncol. 2025 Nov 21;33(3):2674–82. doi: 10.1245/s10434-025-18698-4 (PMC12901085; doi:10.1245/s10434-025-18698-4)
Supplement: Supplementary file 1 — Supplementary file1 (DOCX 25 KB) [file 10434_2025_18698_MOESM1_ESM.docx]

**Suppl. Table 1**: Neoadjuvant chemotherapy regimen.

| n (%) | Total | Folfirinox | Gemcitabine / nab-Paclitaxel | Other | p-value |  |
| --- | --- | --- | --- | --- | --- | --- |
|  | **(n=310)** | **(n=247)** | **(n=33)** | **(n=30)** |  | |
| Chyle leak |  |  |  |  |  | |
| Grade 0 | 197 (63.6) | 153 (61.9) | 24 (75.0) | 20 (66.7) | 0.8358 | |
| Grade A | 69 (22.3) | 59 (23.9) | 4 (12.1) | 6 (20.0) |  |  |
| Grade B | 33 (10.6) | 26 (10.5) | 4 (12.1) | 3 (10.0) |  |  |
| Grade C | 11 (3.5) | 9 (3.6) | 1 (3.0) | 1 (3.3) |  |  |
| Grade B/C | 44 (14.2) | 35 (14.2) | 5 (15.2) | 4 (13.3) | 0.9555 | |

*Legend: FOLFIRINOX; 5-Fluorouracil, leucovorin, irinotecan, oxaliplatin, Gemcitabine/nab-Paclitaxel; Combination chemotherapy protocol.*

**Suppl. Table 2:** Univariable analysis of factors potentially associated with cyle leak grade A-C.

| Parameter | N | Chyle leak grade A-C (%) | Odds ratio | 95% confidence interval | p-value |  |
| --- | --- | --- | --- | --- | --- | --- |
| Total | **1402** | **407 (29.0)** |  |  |  | |
| Age (years) |  |  |  |  | **0.0293** | |
| - <70 | 861 | 268 (31.1) | 1 | - |  | |
| - ≥70 | 541 | 139 (25.7) | 0.77 | 0.60 – 0.97 |  | |
| Gender |  |  |  |  | 0.6309 | |
| - male | 730 | 216 (29.6) | 1 | - |  | |
| - female | 672 | 191 (28.4) | 0.95 | 0.75 – 1.19 |  | |
| ASA |  |  |  |  | 0.1152 | |
| - ASA 1/2 | 826 | 228 (27.6) | 1 | - |  | |
| - ASA 3/4 | 499 | 158 (31.7) | 1.22 | 0.95 – 1.55 |  | |
| Diabetes |  |  |  |  | 0.0637 | |
| - no | 1005 | 276 (27.5) | 1 | - |  | |
| - yes | 339 | 111 (32.7) | 1.29 | 0.98 – 1.68 |  | |
| Jaundice |  |  |  |  | 0.6137 | |
| - no | 619 | 172 (27.8) | 1 | - |  | |
| - yes | 537 | 157 (29.1) | 1.07 | 0.83 – 1.38 |  | |
| Neoadjuvant chemotheray |  |  |  |  | **0.0016** | |
| - no | 1090 | 295 (27.0) | 1 | - |  | |
| - yes | 312 | 113 (36.2) | 1.54 | 1.18 – 2.01 |  | |
| Type of surgery |  |  |  |  | **<0.0001** | |
| - DP | 317 | 81 (25.6) | 1 | - |  | |
| - PD | 718 | 168 (23.4) | 0.89 | 0.66 – 1.21 |  | |
| - TP | 367 | 158 (43.1) | 2.21 | 1.60 – 3.07 |  | |
| Type of resection |  |  |  |  | **0.0012** | |
| - 1 | 654 | 164 (25.1) | 1 | - |  | |
| - 2 | 359 | 105 (29.3) | 1.24 | 0.93 – 1.65 |  | |
| - 3 | 280 | 92 (32.9) | 1.46 | 1.08 – 1.98 |  | |
| - 4 | 109 | 46 (42.2) | 2.18 | 1.43 – 3.31 |  | |
| Tumor size status |  |  |  |  | 0.0714 | |
| - 8^th^ pT0 | 12 | 4 (33.3) | 1 | - |  | |
| - 8^th^ pT1 | 138 | 30 (21.7) | 1 | - |  | |
| - 8^th^ pT2 | 756 | 210 (27.8) | 1.31 | 0.88 – 2.01 |  | |
| - 8^th^ pT3 | 466 | 153 (32.8) | 1.67 | 1.10 – 2.59 |  | |
| - 8^th^ pT4 | 30 | 10 (33.3) | 1.71 | 0.71 – 3.93 |  | |
| Positive node count |  |  |  |  | 0.5461 | |
| - 0 | 328 | 88 (26.8) | 1 | - |  | |
| - 1-3 | 487 | 148 (30.4) | 1.19 | 0.87 – 1.63 |  | |
| - ≥ 4 | 587 | 171 (29.1) | 1.12 | 0.83 – 1.52 |  | |
| Lymph node ratio |  |  |  |  | 0.2922 | |
| - 0 | 328 | 88 (26.8) | 1 | - |  | |
| - >0 - <0.1 | 413 | 134 (32.5) | 1.31 | 0.95 – 1.81 |  | |
| - 0.1 - <0.2 | 307 | 89 (29.0) | 1.11 | 0.79 – 1.58 |  | |
| - ≥0.2 | 354 | 96 (27.1) | 1.02 | 0.72 – 1.43 |  | |
| Distant metastasis |  |  |  |  | 0.1253 | |
| - cM0 | 1278 | 362 (28.3) | 1 | - |  | |
| - pM1 | 129 | 45 (34.9) | 1.35 | 0.91 – 1.97 |  | |

*Legend: ASA American Society of Anesthesiologists, DP Distal pancreatectomy, PD Pancreatoduodenectomy, TP Total pancreatectomy, pT0–pT4 Pathological tumor stage (8th edition), LNR Lymph node ratio.*
