# Supplementary figures and images for: Neoadjuvant Treatment is a Risk Factor for Clinically Relevant Chyle Leak (ISGPS Grade B/C) After Pancreatic Cancer Resection: A Retrospective Cohort Study
Source: Ann Surg Oncol. 2025 Nov 21;33(3):2674–82. doi: 10.1245/s10434-025-18698-4 (PMC12901085; doi:10.1245/s10434-025-18698-4)

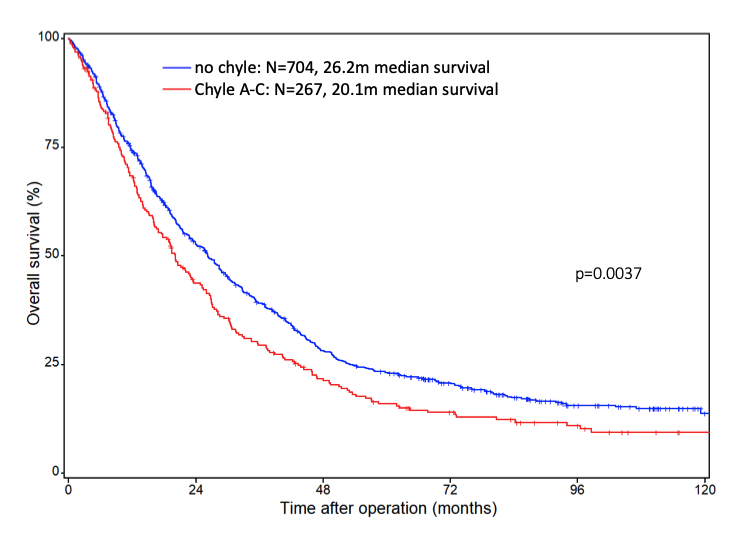

Supplement: Supplementary file 2 — Supplementary file2 (TIFF 1602 KB) [file 10434_2025_18698_MOESM2_ESM.tiff]

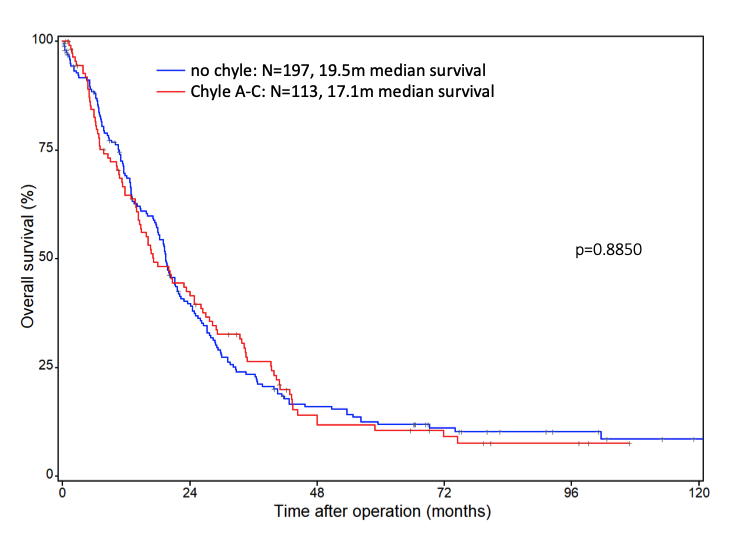

Supplement: Supplementary file 3 — Supplementary file3 (TIFF 1609 KB) [file 10434_2025_18698_MOESM3_ESM.tiff]
